# Optical Fibre Sensor for Capillary Refill Time and Contact Pressure Measurements under the Foot

**DOI:** 10.3390/s21186072

**Published:** 2021-09-10

**Authors:** Hattan K. Ballaji, Ricardo Correia, Chong Liu, Serhiy Korposh, Barrie R. Hayes-Gill, Alison Musgrove, Stephen P. Morgan

**Affiliations:** 1 Optics and Photonics Group, Faculty of Engineering, University of Nottingham, Nottingham NG7 2RD, UK; eexhb4@exmail.nottingham.ac.uk (H.K.B.); Ricardo.GoncalvesCorreia@nottingham.ac.uk (R.C.); Chong.Liu1@nottingham.ac.uk (C.L.); s.korposh@nottingham.ac.uk (S.K.); barrie.hayes-gill@nottingham.ac.uk (B.R.H.-G.); 2 Computer Engineering Department, College of Computers and Information System, Umm Al-Qura University, Makkah 24381, Saudi Arabia; 3 Nottingham University Hospitals NHS Trust, Nottingham NG5 1PB, UK; alison.musgrove@nuh.nhs.uk

**Keywords:** capillary refill time, optical fibre, plastic optical fibre, photoplethysmography (PPG), fibre Bragg grating (FBG), blood volume changes, contact pressure, peripheral arterial disease

## Abstract

Capillary refill time (CRT) refers to the time taken for body tissue to regain its colour after an applied blanching pressure is released. Usually, pressure is manually applied and not measured. Upon release of pressure, simple mental counting is typically used to estimate how long it takes for the skin to regain its colour. However, this method is subjective and can provide inaccurate readings due to human error. CRT is often used to assess shock and hydration but also has the potential to assess peripheral arterial disease which can result in tissue breakdown, foot ulcers and ultimately amputation, especially in people with diabetes. The aim of this study was to design an optical fibre sensor to simultaneously detect blood volume changes and the contact pressure applied to the foot. The CRT probe combines two sensors: a plastic optical fibre (POF) based on photoplethysmography (PPG) to measure blood volume changes and a fibre Bragg grating to measure skin contact pressure. The results from 10 healthy volunteers demonstrate that the blanching pressure on the subject’s first metatarsal head of the foot was 100.8 ± 4.8 kPa (mean and standard deviation), the average CRT was 1.37 ± 0.46 s and the time to achieve a stable blood volume was 4.77 ± 1.57 s. For individual volunteers, the fastest CRT measured was 0.82 ± 0.11 and the slowest 1.94 ± 0.49 s. The combined sensor and curve fitting process has the potential to provide increased reliability and accuracy for CRT measurement of the foot in diabetic foot ulcer clinics and in the community.

## 1. Introduction

Capillary refill time (CRT) is the time it takes for a distal capillary bed to regain its colour after blanching caused by an applied pressure [1,2]. Manual application of pressure on the forearm, finger, sole, toes and sternum is most commonly employed in conjunction with mental counting [3]. Three to five seconds is sufficient to blanch an area [4], and normal CRT is approximately 2 s, depending on the body temperature and age [1,5]. However, this approach could lead to inaccurate results due to human error, and automated CRT measurement offers the possibility of improving reliability, reproducibility and accuracy.

Diabetic foot ulcers are an economic burden for healthcare providers, and the cumulative lifetime incidence of non-traumatic foot resection is up to 15.6% [6]. The mortality rate is typically 70% within 5 years after foot resection surgery [7], and the sequela is usually worse than other diseases, such as cancer [8]. Kruse and Edelman suggested that vascular assessment by measuring the CRT from the foot is essential to evaluate tissue breakdown of people with diabetes [9]. In other research [10], manual CRT measurements were demonstrated to be unable to discriminate between patients with and without peripheral arterial disease, although reliability of manual measurement between testers was highlighted as a problem. Therefore, monitoring the CRT from the sole of the foot using an automated device has the potential to be a useful tool in revealing early-stage tissue breakdown. Automated CRT measurement are under development [11,12,13]. Two systems [11,12] did not measure contact pressure, which is known to affect CRT [14,15]. Others [13,14,16] designed an automated CRT device that takes into consideration the applied pressure whilst measuring the reflected light intensity; however, this is based on bulky mechanical and optoelectronic components.

Optical fibre sensors are a highly versatile platform for tissue measurements due to their small footprint, their ability to measure a wide range of physical and physiological parameters and their immunity to electromagnetic interference [17]. Our group previously demonstrated an integrated optical fibre sensor simultaneously measuring the CRT and applied pressure [2]. This design consisted of a photoplethysmography (PPG) sensor that measured CRT and a fibre Bragg grating (FBG) that measured contact pressure. Proof-of-concept measurements were made on the index finger of healthy volunteers. This paper demonstrates a refined system for automated CRT measurements on the sole of the foot using PPG and FBG sensors with a view to the development of a tool for assessing peripheral arterial disease. New parameters for characterising the CRT response of the foot are proposed, implemented and discussed.

## 2. Methodology

### 2.1. Sensor Design

#### 2.1.1. Contact Pressure

An FBG is a periodic modulation of the optical fibre core refractive index over a short length. FBG sensors reflect a light wavelength that shifts due to the difference in strain, in temperature or in both. This phenomenon indicates that a small range of wavelengths are reflected while the others are transmitted [18]. Strain affects the FBG grating period, which can be measured as a shift in the Bragg wavelength (Δλ_B_) [19]. The reflected wavelength of the input light is λ_B_, which is considered a first-order Bragg condition of the grating. λ_B_ is sensitive to strain and temperature and can be expressed by Equation (1).

(1) $$\lambda_{B}=2\cdot \Lambda \cdot \eta_{\mathrm{eff}}$$

where Λ is the grating period of refraction variation of the FBG, and $\eta_{\mathrm{eff}}$ the effective refractive index of the fibre core [18].

The total wavelength shift caused by isotropic strain and temperature change can be calculated by Equation (2) [20].

(2) $${\Delta \lambda }_{B}=\lambda_{B}\;[(1-P_{e})\;\times \;ℇ+((1-P_{e})\;\times \;\alpha +\xi )\;\times \;\Delta T]$$

where Δλ_B_ is the total wavelength shift, *P_e_* is the photoelastic constant, ℇ is the isotropic strain, α is the thermal expansion coefficient, ξ is the thermo-optic coefficient of the optical fibre, and ΔT is the temperature variance.

FBGs are most frequently used to measure strain; however, they can be adapted to monitor contact pressure. The principle of operation is that a transverse load is transduced into an axial strain on the fibre. With a bare optical fibre, this is an insensitive method and but has been used to detect high pressure >50 GPa [21,22] in heavy industrial processes, such as geo-technical testing. However, to blanch tissue, a much lower pressure is required (<200 kPa), and it is necessary to increase the pressure sensitivity. Correia et al. encased the FBG in an epoxy layer (with Young’s modulus lower than the optical fibre), which increases the axial strain sensitivity to transverse load [23,24]. As the FBG is also sensitive to strain and temperature, it is essential to eliminate cross sensitivity [25]. This can be achieved by anchoring the fibre to ensure strain is minimised and using an additional FBG as a temperature reference.

**Figure 1:**
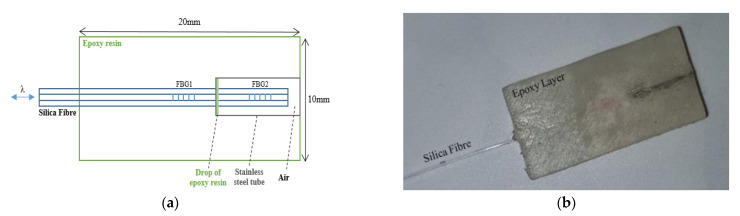
(**a**) Contact pressure sensor schematic, and (**b**) photograph of the final constructed sensor.

Figure 1 shows the contact pressure sensor schematic (Figure 1a) and a photograph (Figure 1b). Two FBGs with Bragg wavelengths of 1537 nm (FBG1) and 1546 nm (FBG2) were inscribed in a 125 µm diameter photosensitive silica fibre (PS1250 Fibercore Ltd., Southampton, UK) using a UV laser and a phase mask [23,24]. The distance between the FBG centres was 8 mm and the length of each was 3 mm. In order to isolate FBG2 from the applied pressure, it was covered by a stainless-steel tube (inner diameter: 0.3 mm, outer diameter: 0.5 mm, Coopers Needle Works Ltd., Birmingham, UK) and anchored on the side near FBG1 using a small drop of epoxy (Vitralit 1655, Panacol-Elosol GmbH, Steinbach, Germany). The end of the silica fibre located inside the stainless-steel tube was not anchored and was free from strain; hence, FBG2 can be used as a temperature reference to compensate FBG1.

Using an appropriate mould and UV curing for 20 min, the two FBGs were embedded into an epoxy layer (Vitralit 1655) to raise the contact pressure sensitivity. This enabled typical tissue blanching pressures <200 kPa to be measured with FBG1. The sensor size was W = 10 mm × L = 20 mm × H = 2 mm which allowed it to be positioned over the first metatarsal head of the foot.

#### 2.1.2. PPG Sensor

**Figure 2:**
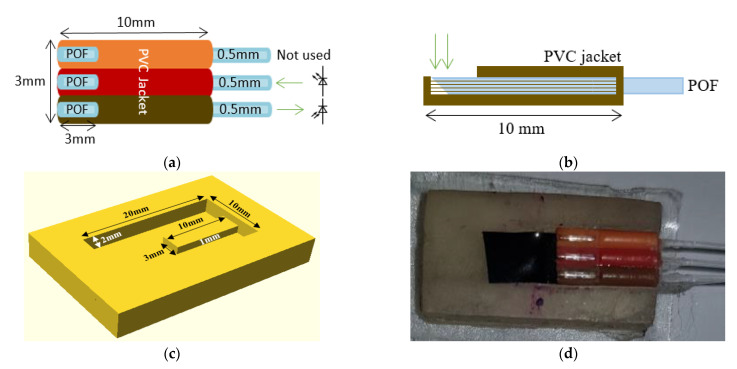
(**a**) PPG sensor for measuring changes in blood volume. The POF brown (receives light) and POF red (transmits light) channels are only used in this application. (**b**) Side view of PPG sensor. (**c**) 3D mould for embedding PPG sensor in an epoxy layer. (**d**) Photograph of the PPG sensor integrated within the epoxy layer used for CRT measurements.

A PPG sensor was constructed to measure the blood volume changes (Figure 2). This was described previously [26] and consisted of three plastic optical fibres (POFs) with the distal end of the fibres cleaved at 45° to increase the amount of light directly transmitted onto the skin at 90° [27]. The first POF (brown channel) was used to receive light, the second POF (red channel) was used to transmit light, and the third POF (orange channel) was not used in this study, although it could be used in future for additional wavelengths. The cleaved end of the three fibres were fixed inside three 10 mm long PVC jackets [26]. Three millimetres were removed from one side of the PVC jacket to expose the fibres where they would touch the skin, as shown in Figure 2a in plan view and Figure 2b in side view. The PPG sensor was embedded within a UV-cured epoxy layer (10 mm × 20 mm × 2 mm) using a mould (Figure 2c). The cured layer accommodated the 3 mm × 10 mm × 1 mm PPG sensor (from Figure 2a) in a slot. The slot was covered by black tape before fixing the sensor to eliminate light from travelling into the layer, which could have caused light shunting between the layer and the received fibre. Figure 2d shows a photograph of the assembled PPG sensor integrated within the epoxy layer.

#### 2.1.3. Combined Sensor for Contact Pressure and CRT

**Figure 3:**
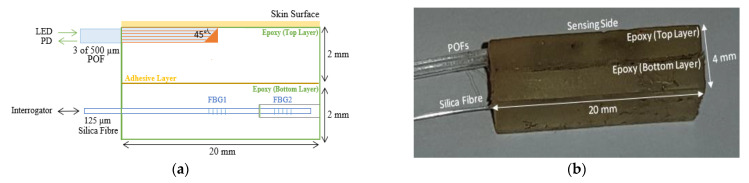
(**a**) Side view schematic of the combined sensor of the CRT measurements (PPG and contact pressure) (LED—light emitting diode, POF—plastic optical fibre, FBG—fibre Bragg grating, PD—photodiode) (**b**) Photograph of the sensor.

The sensors described in the previous two sections were combined using a thin layer of epoxy. Figure 3a shows a schematic of the side view of the combined sensor for measuring the blood volume changes (top layer) and the contact pressure (bottom layer) simultaneously, and Figure 3b shows a photograph of the constructed sensor.

### 2.2. Experiments

#### 2.2.1. Temperature Calibration of the FBG Sensors

Both FBGs are thermally calibrated so that FBG2 can be used to compensate FBG1. The three main pieces of equipment used were an oven (Binder, ED115, Tuttlingen, Germany) to control the temperature, a SmartScope interrogator (Smartfibres, Bracknell, UK) to interrogate and measure the wavelength shift of both FBGs and a thermocouple data logger (PICO TC-08, Type K, St Neots, UK) to record the temperature (type K thermocouple, accuracy = 0.5 °C).

The sensor was fixed by heat resistant tape to a glass plate inside the oven, and the thermocouple was close by. The optical fibre sensor and thermocouple connections passed through an access port on the oven and were connected to the interrogator and data logger, respectively. Both measurement units were connected to a computer through USB cables.

Temperature was continuously increased from room temperature (~25 °C) to ~50 °C over a period of 15 min. The temperature was then held constant at ~50 °C for a further 15 min before being allowed to cool down naturally to room temperature by opening the oven door. During calibration the interrogator measures the peak wavelength of the two FBGs and the results were compared with the thermocouple data.

#### 2.2.2. Contact Pressure Calibration

**Figure 4:**
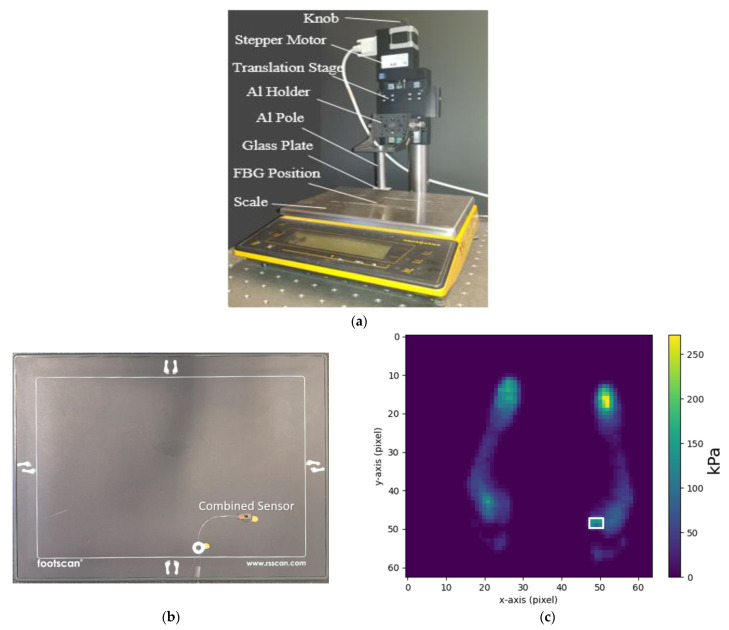
(**a**) Pressure calibration system comprising a stepper motor and scale to apply a known force to the FBGs. (**b**) Photograph of the force measurement plate (RS Scan Footscan) with the combined sensor on top of it. The active area of the foot scan plate was 488 mm × 325 mm with 4096 sensors (arranged in a 64 × 64 matrix). The dimension of each foot scan plate sensor (pixel) was 7.62 mm × 5.08 mm. (**c**) Pressure map under the foot. The location of the combined optical fibre sensor underfoot is highlighted in the white frame.

Figure 4 shows the loading set-up deployed for contact pressure calibration. The applied pressure was controlled by a stepper motor (C-663 Mercury Step controller with M-403.22S precision translation stage, PI, Karlsruhe, Germany) which lowered and raised an aluminium (Al) pole attached to a glass plate. The contact pressure sensor was placed on a scale (Sartorius MC1 LC 4800 P, Goettingen, Germany) and under a glass plate so that force and wavelength shift could be obtained simultaneously with data recorded on a PC.

For calibration, and to assess reliability and repeatability of the contact pressure sensor, the applied pressure was cycled between zero and approximately 100 kPa three times in a stepwise manner (pressure increment = 14 kPa, duration = 30 s).

Foot pressure measurements were also conducted using a commercially available plantar pressure measurement system (Footscan, RS Scan Ltd., Ipswich, UK) that provides a spatial map of force under the foot. The optical fibre pressure sensor was fixed onto the surface of the measurement plate (Figure 4b), and three healthy volunteers (mass 67 kg, 69 kg, 79 kg) repeatedly stepped on and off the sensor at the first metatarsal head. The area of the optical fibre sensor was identified on the measurement plate so that the force measurements could be converted into pressure, allowing a direct comparison of the two sensors via Bland–Altman analysis.

#### 2.2.3. CRT and Contact Pressure Measurements

The aim of the experiment was to investigate the feasibility of reliably making measurements under the foot, initially using healthy volunteers. The light source deployed for CRT/PPG measurement was a green LED (λ = 530 nm, M530F1, Thorlabs, NJ, USA). Green light is chosen as this has previously been demonstrated [15] to provide higher contrast to capillary refill signals than red or near infrared wavelengths due to the higher absorption of blood in this range. The typical output power from a 400 µm diameter POF coupling the LED was 9.9 mW. The detector was a photodiode (PDA36A, Thorlabs, NJ, USA) with a wavelength range of 350–1100 nm with the photocurrent passed to a transimpedance amplifier (TIA). The output voltage from the TIA was connected to a data acquisition card (DAQ, myDAQ, 16 bits, National Instruments (NI), Austin, TX, USA) with data recorded on a PC. The gain of the transimpedance amplifier (TIA) and the spectral responsivity (R_λ_) of the photodiode were 4.75 × 10^6^ V/A and 0.19 A/W (at λ = 530 nm), respectively. These values were used to convert the TIA output from voltage to power. A SmartScope interrogator (Smartfibres, Bracknell, UK) was again used to measure the wavelength shift and hence pressure exerted on the FBG sensor.

**Figure 5:**
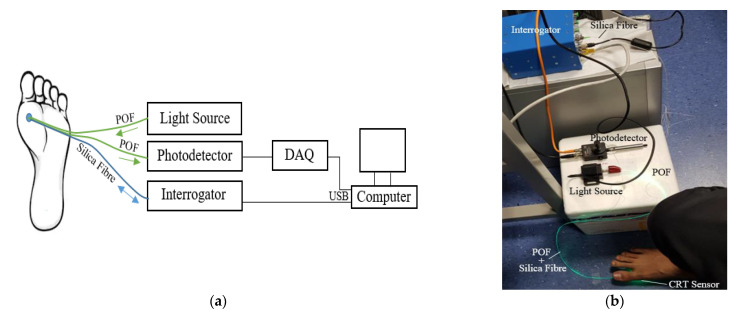
Schematic (**a**) and photograph (**b**) of the experimental setup of the CRT and contact pressure measurements; light source (LED, λ = 530 nm), photodetector (PD + TIA) PDA36A, interrogator (SmartScope), DAQ–data acquisition card. The sensor sits under the foot of the volunteer.

Figure 5a shows a schematic of the experimental setup and the position under the foot investigated. The combined sensor (Figure 3) was placed on the floor, and the first metatarsal head of the foot was placed on the sensor. Green light was transmitted through the POF to the skin, and the reflected light was collected by the second POF and delivered to the photodiode. In parallel with this the output from the interrogator was connected to the SmartScope software on the computer via a USB connection. Figure 5b shows a photograph of the experimental setup of the CRT measurements. The final collected data of the CRT/PPG reflected light intensity, and the FBG contact pressure changes (measured by POF and the silica FBG fibre respectively) were then subsequently analysed offline by MATLAB R2018b.

The sensor (Figure 3) was fixed on the floor, and the thermocouple was placed on top of the sensor to measure the underfoot temperature. At the beginning of the experiment, the participants were asked to sit still for fifteen minutes to relax and acclimatise to the room environment. After this initial period, the participant’s right foot was placed in contact with the sensor but without deliberately applying any pressure.

Contact pressure, PPG and underfoot temperature signals were then recorded simultaneously for 10 cycles of applying (10 s) and releasing (10 s) pressure. Each participant pressed the foot on the sensing layer until no pulsatile signals were visible. The 10 s delay between each cycle was determined empirically prior to formal experiments by investigating the typical time to return to baseline.

### 2.3. Signal Processing

**Figure 6:**
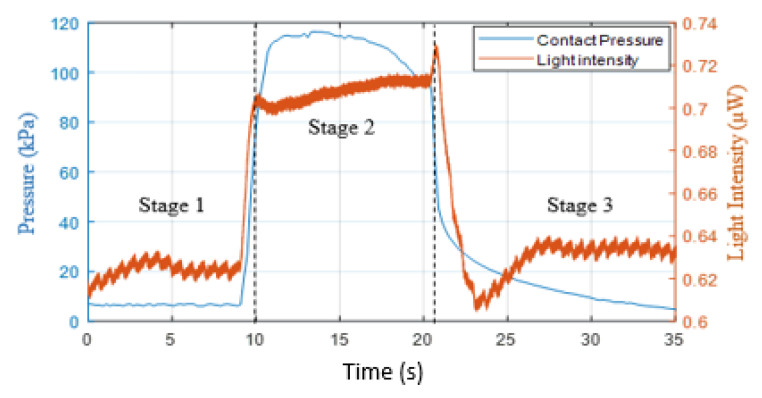
Recording of the changes of the PPG (orange trace) and the skin contact pressure (blue trace) simultaneously. The normal stage was approximately 0 to 10 s; the applying pressure stage was 10 to 21 s, and the refilling stage started after 21 s.

The process involved extracting, normalising and fitting of the refilling signal to estimate the capillary refill time. An example of a typical measurement from under the foot is shown in Figure 6. The reflected light intensity was inversely proportional to blood volume. The figure presents three stages of measuring the blood volume changes: normal (0–10 s), applying pressure (10–21 s) and refilling (21 s). The PPG recorded changes in light intensity of the foot’s sole by applying pressure (orange trace) whilst the FBG recorded the skin contact pressure (blue trace). Initially, the foot was in contact with the sensor but with no weight deliberately applied (~5 kPa, Stage one). After 10 s, pressure was applied on the sensor, which was sufficient (~115 kPa) to evacuate blood from the capillary bed, which increased the DC light level and suppressed the pulsatile signal (Stage 2, 10–21 s). At approximately 21 s, the applied pressure was released, and the blood started again to flow in the capillary bed; hence, the pulsatile signal re-appeared (Stage 3), as shown in Figure 6. This final stage (refilling stage) was only required to calculate the CRT. The contact pressure sharply decreased when the blood started to refill the capillary bed from ~95 to ~5 kPa. It is interesting to note that the PPG signal reduced below the baseline due to post-occlusion reactive hyperaemia and the pulsatile signal gradually returned.

CRT is typically defined as the time taken for the intensity signal to change from 90% to 10% of its maximum level [2]. Calculating the CRT directly from the complete refilling signal (i.e., Stage 3, in Figure 6) has some limitations, as the DC baseline varies due to respiration, vasoconstrictor waves and Mayer waves [28] and motion artefacts can distort the signal. To reduce the impact of these issues on the CRT measurements, a normalising and fitting process was applied on the refill signal [2,14].

The refilling signal was normalised within the range 0–1 [29]:

(3) $$I_{n}=\frac{I_{o}-I_{min}}{I_{max}-I_{min}}$$

where *I_n_* is the normalised light intensity of the refilling signal, *I_o_* is the original light intensity of the refilling signal, *I_min_* is the minimum value of the refilling signal, and *I_max_* is the maximum value of the refilling signal.

**Figure 7:**
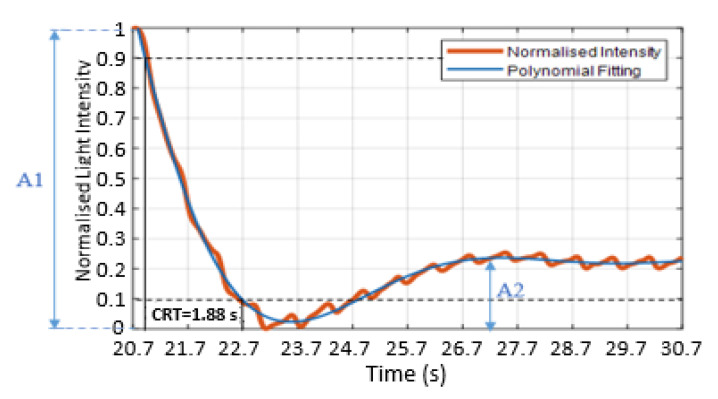
Normalised refilling signals (orange trace) with the polynomial curve fitting (blue trace). Estimating the CRT used the maximum and minimum thresholds method. The maximum threshold is 0.9 (black dashed line), and the minimum threshold is 0.1 (black dashed line). The CRT is the calculated time of the refilling signal between 0.9 and 0.1. A1 is the amplitude of the blanching intensity relative to the starting intensity, and A2 is the amplitude of the minimum intensity to the next local maximum.

Liu et al. [2] and Kviesis-Kipge et al. [30] applied a fitting process that depended on splitting the refilling signal into the blood refilling period and the baseline period. The blood refilling period was when the light intensity decreased significantly at the beginning of blood returning to the capillaries. The baseline period was when the pulsatile signal returned after blanching. The authors used exponential fitting and straight linear fitting for estimating the CRT [2,30]. This method of fitting works efficiently when the baseline region has a stable pulsatile signal. However, if the signal has fluctuations on the baseline region, as shown in Figure 7 (orange trace), which are caused by post-occlusion reactive hyperaemia [31] (22.7–24.7 s) (which could be informative about the skin properties), then straight line fitting is not accurate. Therefore, in this paper, a seventh-order polynomial fitting was applied:

(4) $$I_{fn\left(poly.\right)}=c_{0}+c_{1}x+c_{2}x^{2}+c_{3}x^{3}+c_{4}x^{4}+c_{5}x^{5}+c_{6}x^{6}+c_{7}x^{7}$$

where *I_fn(poly.)_* is the fitted signal of the normalised light intensity using the polynomial fitting, and “c” is a set of coefficients. The R^2^ of the curve fitting increased as the order of the polynomial increased, with an order of 7 providing good fits for all measured data. As an example, for volunteer 8, the R^2^ value was typically 0.86 for order 2, 0.91 for order 5 and 0.97 for order 7.

Figure 7 presents the polynomial fitting signal (blue trace) of the normalised refill light intensity (orange trace). The fitting curve was assessed based on the root-mean-square error (RMSE) of the fit and R^2^. Figure 7 is well matched to the refilling signal and the curve fitting with RMSE = 0.016 (normalised units) and $R^{2}=0.99,$ whilst the fitting was able to plot a good fit through the pulsatile signals.

The CRT was estimated as the time taken for the normalised signal to transition from 0.9 to 0.1 [2,32,33]. Figure 7 shows the maximum threshold at 0.9 (upper black dashed line) and the minimum threshold at 0.1 (lower black dashed line) and the CRT (solid vertical lines), which in this case is 1.88 s. The sharp drop of the refilling signal and the first peak after the post-occlusive reactive hyperaemia period (22.7–24.7 s) were defined as the amplitude of the blanching intensity relative to the starting intensity (A1) and the amplitude of the minimum intensity to next local maximum (A2), respectively, as shown in Figure 7.

In order to quantify the refilling signals quality, two features were measured—namely, perfusion index (PI) and average peak-to-peak (P–P)_ave_ interval. The PI was calculated as discussed by Ballaji et al. [26], which is PI (%) = (AC/DC) × 100. The (P–P)_ave_ interval was the distance between two sequential PPG peaks, which represents a completed heart cycle [34,35,36].

## 3. Results and Discussion

### 3.1. Temperature Response of the Contact Pressure Sensor

**Figure 8:**
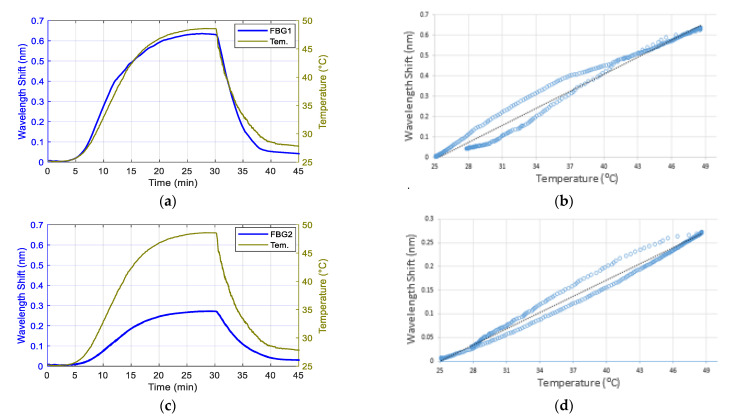
The temperature responses of FBG1 (**a**) and FBG2 (**c**) sensors within the epoxy layer; the green traces show the temperature changes measured with a thermocouple and the blue trace presents the wavelength shift peak of FBGs. (**b**) and (**d**) are the hysteresis diagrams of the wavelength shift of FBG1 (**b**) and FBG2 (**d**) against variable temperatures.

Figure 8a and c shows the temperature response of FBG1 and FBG2. Figure 8b,d show the hysteresis diagrams of the wavelength shifts of the two FBGs against temperature changes with a near linear relationship. Using linear fitting for FBG1 and FBG2 temperature responses were ≈27.9 pm/°C (R^2^ = 0.98) and 11.4 pm/°C (R^2^ = 0.99), respectively. The temperature equations of both FBGs were deduced from the linear fitting equations as follows:

(5) $$T_{1}\;=\;35.84\;\times \;\Delta \lambda_{\mathrm{FBG}1}\;+\;25.35$$

(6) $$T_{2}\;=\;87.72\;\times \;\Delta \lambda_{\mathrm{FBG}2}\;+\;24.87$$

T_1_ and T_2_ are the temperature (°C), and Δλ_FBG1_ and Δλ_FBG2_ are the wavelength shifts of FBG1 and FBG2, respectively.

The temperature sensitivity of FBG1 was higher than FBG2 because FBG1 was embedded and in contact with the epoxy whereas FBG2 was encased inside a stainless-steel tube, as shown in Figure 1. According to Mihailov, it is important to consider the effect of the material temperature when encasing the FBG sensor with a bonding material to increase the pressure sensitivity of the FBG using [37]:

(7) $$\frac{{\Delta \lambda }_{B}}{\lambda_{B}}\;\mathrm{Pe}\;\times \;\varepsilon \;+\;[\mathrm{Pe}\;\times \;(a_{s}\;-\;a_{f}\;)\;+\;\zeta ]\;\times \;\Delta T$$

where ${\Delta \lambda }_{B}$ is the Bragg wavelength shift, λ_B_ is the Bragg wavelength, P_e_ is the strain-optic coefficient, ε is the strain, a_s_ is the coefficient of thermal expansion (CTE) of a fibre bonding material, a_f_ is the CTE of the fibre itself, ζ is the thermo-optic coefficient, and ΔT is the temperature change. As the CTE of the epoxy (214 ppm/K [38]) was higher than the CTE of the silica fibre (2.6 ppm/K [39]), the temperature sensitivity of FBG1 was higher than FBG2.

**Figure 9:**
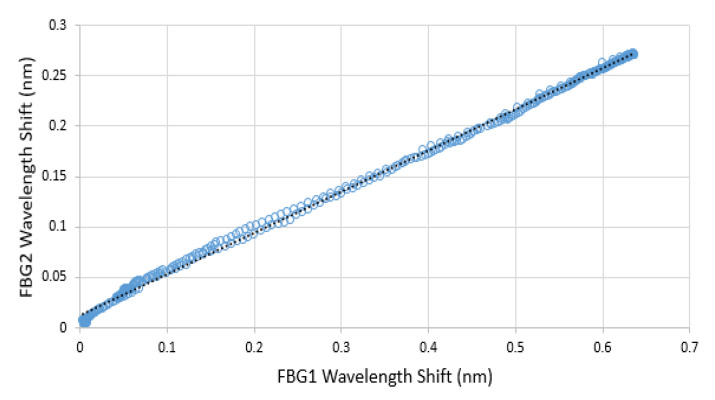
The wavelength shift of FBG1 against the wavelength shift of FBG2 with increase and decrease of temperature.

Although FBG1 had higher temperature sensitivity than FBG2, there was a linear relationship (R^2^ = 0.9986) between them during the temperature changes, as shown in Figure 9. Therefore, for the FBGs embedded within the epoxy layer, it was therefore possible to infer the wavelength shift of FBG1 due to its temperature change by utilizing the wavelength shift of FBG2:

(8) $$\Delta \lambda_{T\text{\_}\mathrm{FBG}1}\;=\;2.44\;\times \;\Delta \lambda_{\mathrm{FBG}1}\;-\;0.03$$

where Δλ_T_FBG1_ is the wavelength shift of FBG1 due to temperature, and Δλ_FBG2_ is the wavelength shift of FBG2.

Equation (8) was therefore used to compensate the temperature changes in FBG1 to enable applied contact pressure to be measured.

### 3.2. Response of the Contact Pressure Sensor to Applied Loads

The synchronised FBG2 wavelength shift was subsequently applied to compensate for the effect of room temperature:

Δλ_P_FBG1_ = Δλ_FBG1_ − Δλ_T_FBG1_ (9)

where Δλ_P_FBG1_ is the wavelength shift of FBG1 due to pressure alone, Δλ_FBG1_ is the wavelength shifts of the contact pressure sensor, and Δλ_T_FBG1_ is the wavelength shift due to temperature obtained from Equation (8).

**Figure 10:**
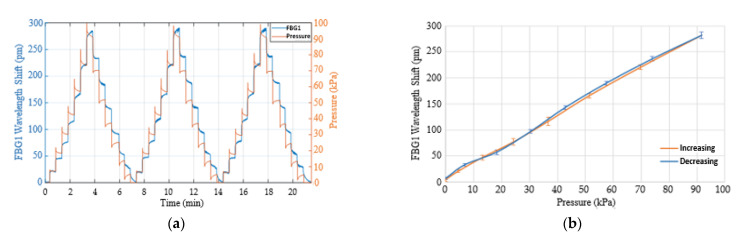
(**a**) The loaded and unloaded pressure steps of FBG1 sensor after compensating for temperature. (**b**) FBG1 wavelength shift against loaded and unloaded pressure.

Figure 10a shows the stepped loaded and unloaded pressure response after compensating for temperature. There was a clear difference between pressure measured by the weighing scale (orange trace) and Δλ_P_FBG1_ (blue trace) when pressure decreased, which was caused by the epoxy material which needs some time to return to the original shape. However, the average relationship between the increasing and decreasing pressure and the wavelength shifts of the FBG1 is shown in Figure 10b. The results indicate a linear relationship between the applied pressure and the wavelength shift, with an R^2^ of 0.998. The pressure (P) is deduced from the linear relationship:

(10) $$P\;(\mathrm{kPa})=0.328\times \;\Delta \lambda_{P\text{\_}\mathrm{FBG}1}-2.433$$

**Figure 11:**
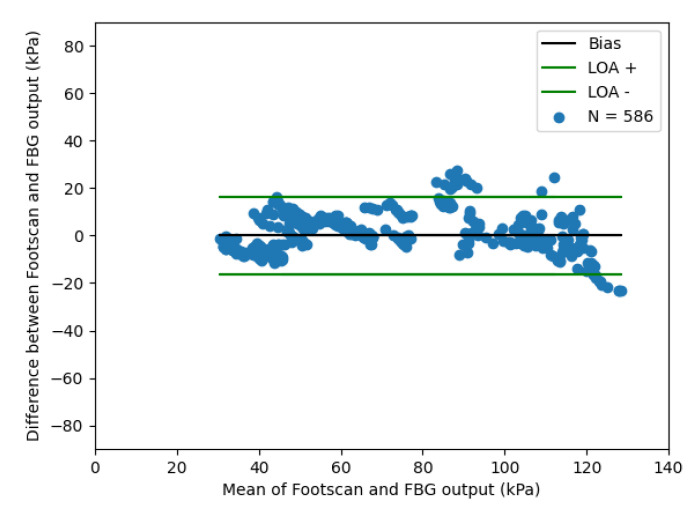
Bland–Altman plot comparing simultaneous response of optical fibre pressure with commercially available plantar pressure measurement device (Footscan). Three volunteers repeatedly stepped on the optical fibre sensor and Footscan. (LOA—limit of agreement).

A Bland–Altman plot of the optical fibre sensor compared with the Footscan device is shown in Figure 11, which demonstrates a limit of agreement of ±20 KPa.

### 3.3. Capillary Refill Time Measurement on the Sole of the Foot

Testing on 10 healthy volunteers was approved by the Ethical Review Committee of the Faculty of Engineering at the University of Nottingham. The 10 healthy participants did not suffer from any known circulatory system diseases. The genders were 7 males and 3 females, with mean ± SD of age, weight and foot (shoe) size of 28.6 ± 3.6 years, 83.3 ± 19.7 kg and 7.8 ± 1.8 UK size, respectively.

**Figure 12:**
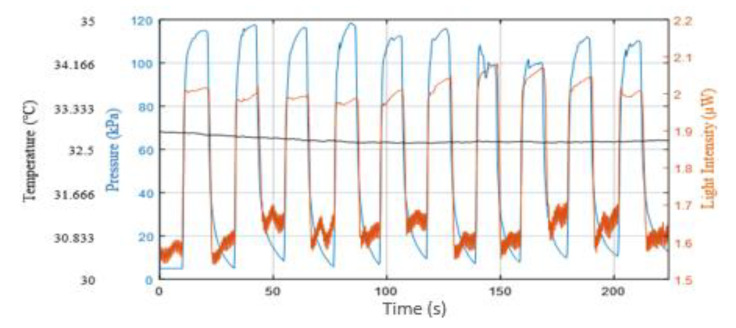
Applied pressure (blue trace), reflected light intensity changes (orange trace) and underfoot temperature (black trace) of one participant. The data were recorded from the contact pressure sensor, the PPG (light intensity) and data logger, respectively. Application and release of pressure was repeated ten times.

**Figure A1:**
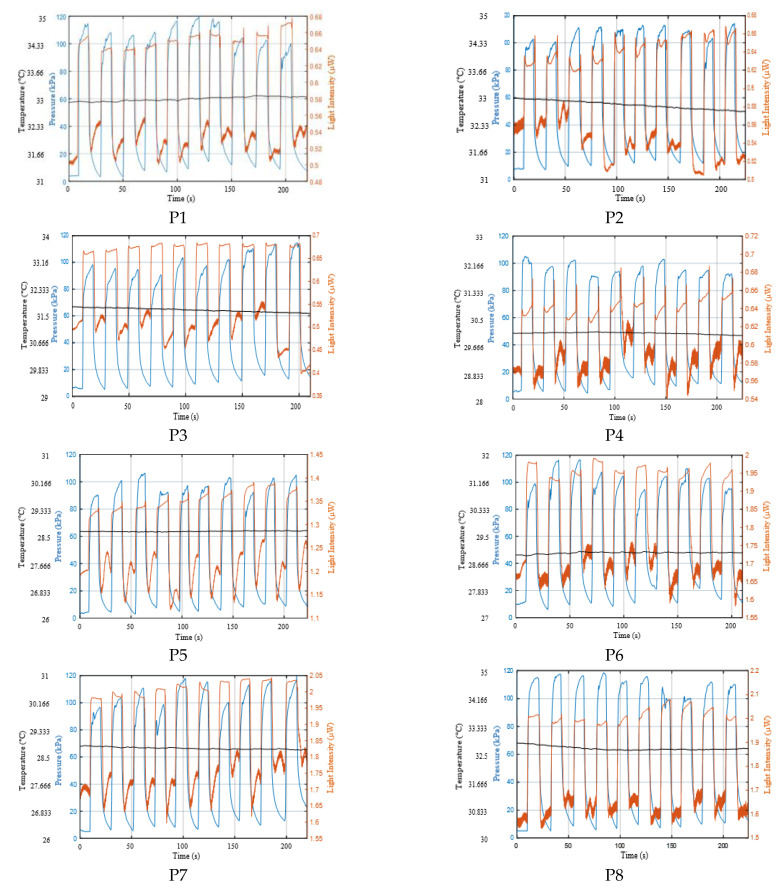

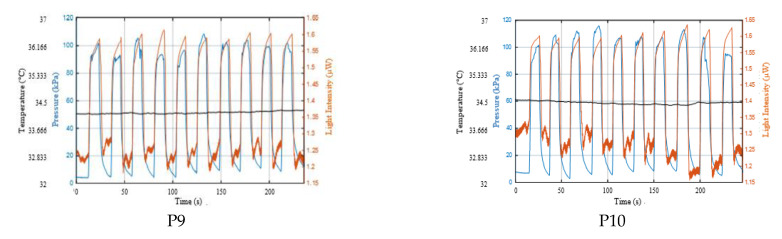
Applied pressure (blue trace), reflected light intensity changes (orange trace) and underfoot temperature (black trace) of ten participants (P1–P10).

Figure 12 shows a typical sensor response for a single participant which shows the applied pressure (blue trace), the reflected light intensity (orange trace) and the underfoot temperature (black trace) with a relatively stable temperature of 32.7 °C. The complete set of participants’ data appears in Appendix A.1 (Figure A1).

**Figure 13:**
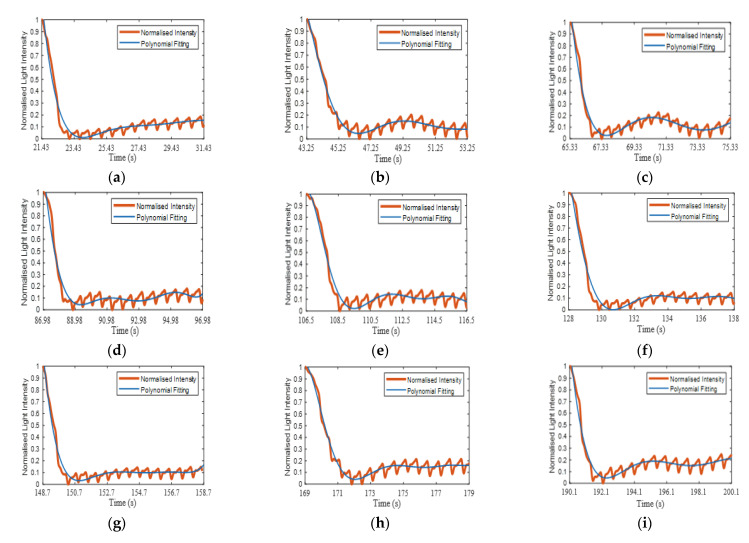

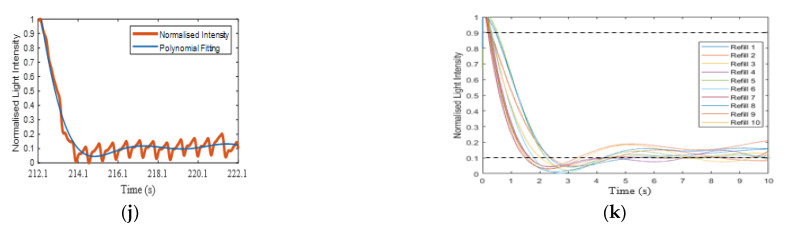
(**a**–**j**) Ten normalised refilling curves (orange) and curve fit (blue) for a single subject. (**k**) Shows the 10 refilling fitted curves for a single subject along with the maximum (0.9) and minimum (0.1) thresholds (black dashed lines).

**Figure A2:**
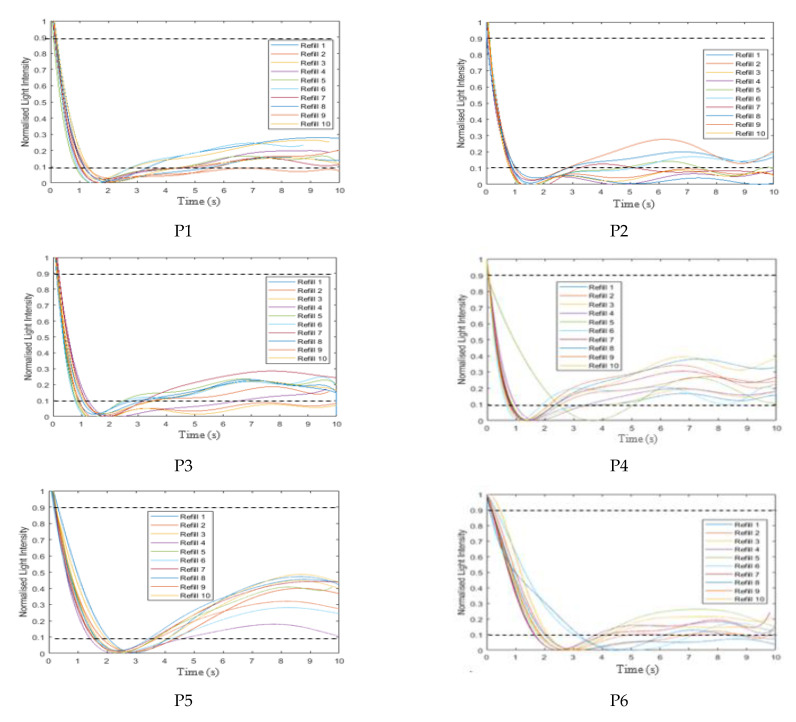

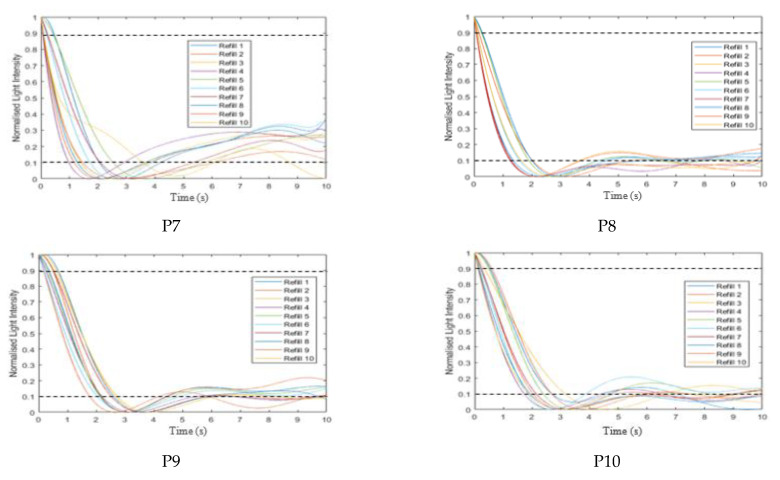
The 10 curves fitting each participant (P1–P10), with the maximum (0.9) and minimum (0.1) thresholds in black dashed lines.

Each refilling phase passed through the signal processing of extracting, normalising, fitting the refilling signal and estimating the CRT, as described in Section 2.3. Figure 13a–j illustrates a close-up of the 10 normalised refilling curves (orange traces) with curve fitting (blue traces). Figure 13k represents all 10 curve fitting traces with the maximum (0.9) and minimum (0.1) thresholds (dotted line). The complete set of the ten volunteers’ curve fitting traces are presented in Appendix A.2 (Figure A2).

Table 1 represents the estimated CRT values with RMSE, R^2^ and C_0_ of the 10 refilling periods, which were calculated from the 10 fitting curves shown in Figure 12. C_0_ is useful, as it is proportional to the estimated CRT value as discussed later in this section. The average CRT of this participant was 1.55 ± 0.28 s, with a RMSE of 0.041, a R^2^ of 0.96 and C_0_ = 9.03 × 10^−6^. The ratio (as a%) between the amplitude of the minimum intensity to the next local maximum (A2) and the amplitude of the blanching intensity relative to the starting intensity (A1) is shown, and its average was 13.5 ± 3.5%. In addition, the time to reach a stable blood volume was estimated, which occurred after the peak (A2). The estimation was based on the stability of the PI of the PPG signals. All these features were calculated from the fitting curve, and they are shaded in blue in Table 1. The table also illustrates the average PI and the (P–P)_ave_ interval of each recovered PPG signal; they were calculated from the refilling signals and are shown in the last 2 columns of Table 1. The average PI and (P–P)_ave_ interval of this participant were 2.60 ± 0.32% and 0.67 ± 0.02 s, respectively.

Table 2 shows the 10-subject individual average values of the CRT (overall = 1.38 ± 0.48 s), polynomial coefficient C_0_, R^2^, RMSE (normalised units), the time to reach the stable blood volume and the ratio between the amplitudes A2 and A1. All these features were calculated from the fitting curves and are presented in blue. The Table also shows the PI and (P–P)_ave_ interval values that were calculated from the refilling signals and are presented in columns 8 and 9. The values of the underfoot temperature as well as those from applying and releasing pressure are also shown in this table; these values were measured by the data logger and the FBG sensor, respectively, and they are presented in the last three columns of this table. All these data summarise the 10 refilling signals of all 10 participants.

**Figure 14:**
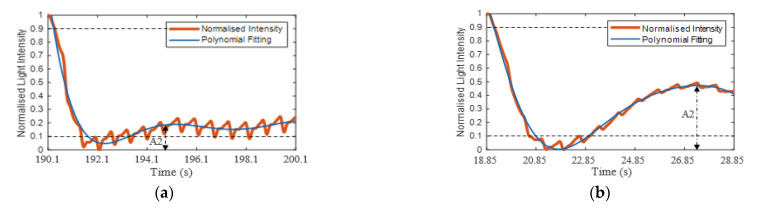
Example of a refilling signal that reached a stable blood volume after (**a**) and before (**b**) passing the first max blood volume to max recovery (A2). The stabilise estimation is based on the stability of the PI of the PPG signals.

Eighty-seven (87%) out of the one hundred refilling signals reached a stable blood volume (based on the stability of the PI of the PPG signals) after passing the first max blood volume to max recovery (A2). However, the rest of the refilling signals (13%) reached a stable blood volume before the A2 point because the refilling signals were gradually increased after the post-occlusion hyperaemia region. For example, Figure 14a and b shows a refilling signal that reached stable blood volume after and before passing A2, respectively.

**Figure 15:**
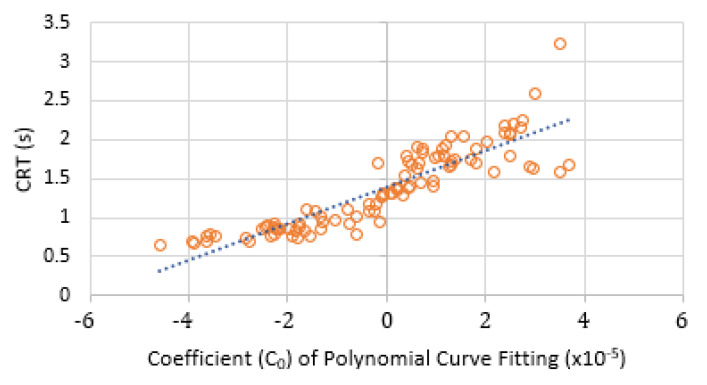
CRT against coefficient C_0_ of the polynomial fitting for all ten volunteers.

The estimated CRT against the coefficient C_0_ of the polynomial fitting were plotted as shown Figure 15. The orange circles show the relationship between the CRT and C_0_. This approximately linear relationship (blue dashed line) offers the potential in the future as a surrogate CRT calculation directly from the polynomial fitting.

The preliminary data presented in this paper are encouraging, and there is an advantage over two previous approaches [13,14], as the system has demonstrated the capability to measure interface pressure alongside the capillary refill signal. Furthermore the system has a relatively small footprint (20 mm × 10 mm × 4 mm) compared with bulkier handheld devices [14]. However, several improvements can be made in future to the system, and it is worth noting limitations in the study design.

Although the sensor is thin (4 mm), it could be incorporated into a level platform, like a conventional weighing scale, to avoid any unnatural distortion of the foot. An alternative is to incorporate the sensor into an insole or textile sock to provide the CRT, PPG and contact pressure in a comfortable and unobtrusive way. This method can also reduce motion artefacts [36] since the sensor will have longer-term contact with the skin. Subsequently, during normal walking, the skin blanching and refilling process can be generated under the foot at every step. The action of walking usually consists of a constant cycle (stance and swing phases) that leads to an approximately constant pressure in terms of the volume and the interval. Therefore, the CRT could be measured accurately during normal walking patterns. Further improvement could also include modifying the size and shape of the probe to allow measurements on the toes. Another limitation is that the sensors are currently manufactured by hand, which affects optical fibre position and polymer layer thickness, and each sensor needs to be individually and periodically calibrated (Figure 10, Equations (9) and (10)). Future work is investigating different approaches such as injection moulding to produce sensors with similar responses.

In future improvements of the opto-electronic unit, the relatively large interrogator could be replaced with a smaller device, such as a miniature FBG interrogator (e.g., Redondo Optics, Inc., Redondo Beach, CA, USA). Here, the dimension of this interrogator is 29 mm × 29 mm × 110 mm and can be battery powered and wireless. The PPG sensor could be miniaturised by replacing the bulky LED and photodiode units with discrete components on a single PCB and using an analogue-to-digital converter chip. Data could be transmitted to the PC wirelessly using a Bluetooth module, and the whole system could be fitted into a small enclosure (estimated size ≈ 120 mm × 50 mm) to make it easy to attach the device to the body for ambulatory monitoring.

The study was conducted with healthy volunteers, and the next stage will be to investigate the use of the sensor in predicting tissue breakdown in those with diabetes. A first study will involve measuring CRT and interface pressure in people with diabetes in collaboration with a multidisciplinary diabetes foot care team. CRT and interface pressure will be measured monthly at points on the feet most likely to develop foot ulcers (e.g., metatarsal heads, heel) with a follow-up investigating whether the measurements could discriminate those who developed diabetic foot ulcers. Although the sensor has been compared with a clinically used plantar pressure measurement device, the study would have benefited from a reference CRT device for validation; however, as noted, the standard clinical approach is to mentally count, which is inadequate. Future work could include the use of cameras to image the CRT for validation.

## 4. Conclusions

This study investigated the design of a new sensor for underfoot CRT and contact pressure measurements. The sensor was used to measure the reflected light intensity changes and the skin contact pressure using a PPG- and FBG-based sensor, respectively. In the CRT measurement, the capillary bed evacuation and refilling processes of blood were performed by applying and releasing the contact pressure. In order to estimate the CRT, the refilling signals passed through four processes—namely, extracting, normalising, fitting the signals and then estimating the CRT values. The perfusion index (PI) and the (P–P)_ave_ interval were calculated from extracting the refilling signals. The normalisation process was applied to minimise the effect of change in the recovery signal. The effect of motion artefacts was reduced by applying a polynomial fitting process, and the time taken for the signal to change from 0.9 to 0.1 of the maximum was used to calculate the CRT of each refilling signal. There were other features extracted from the fitting curve, such as the ratio between the amplitude of the blanching intensity relative to the starting intensity (A1) and the amplitude of the minimum intensity to the local maximum (A2), in addition to the time to reach a stable blood volume. These parameters could be informative for diagnosing peripheral arterial disease.

To the best of the authors’ knowledge, this study is the first to successfully measure the CRT and the contact pressure simultaneously from the sole of the foot. The results of 10 healthy volunteers illustrate that the average applied pressure to blanch and evacuate blood from tissues was 100.76 ± 4.78 kPa. The average CRT was 1.37 ± 0.46 s, and 87% of the refilling signals reached the stable blood volume when passing the first max blood volume after the post-occlusion hyperaemia period. The results also show that there was an approximately linear relationship between the calculated CRT and the coefficient C_0_ of the polynomial fitting. Further investigation of this approximate linear fitting could help to obtain more accurate CRT results directly from the fitting curve.

## Figures and Tables

**Table 1:** Summary CRT results of a single subject with 10 refills. Tabulated here are the RMSE, R^2^, C_0_, CRT, the time to reach the stable blood volume, the ratio between the amplitude of the minimum intensity to the local maximum (A2) and the amplitude of blanching intensity relative to the starting intensity (A1). All these features were calculated from the fitting curves (in blue). Additionally shown are the PI and (P–P)_ave_ interval values, which were calculated from the refilling signals in last 2 columns.

Refill	RMSE	R^2^	C_0_	CRT (s)	Stable Blood Volume (s)	Ratio of A2/A1 (%)	Average PI (%)	(P-P)_ave_ Interval (s)
**1**	0.038	0.95	−6.57 × 10^−7^	1.29	4.77	9	2.08	0.68
**2**	0.036	0.97	2.37 × 10^−5^	1.86	5.96	15	2.65	0.67
**3**	0.040	0.95	7.95 × 10^−7^	1.22	5.07	18	2.33	0.61
**4**	0.049	0.94	2.29 × 10^−6^	1.22	4.26	10	2.49	0.66
**5**	0.046	0.96	1.80 × 10^−5^	1.65	5.15	14	2.51	0.66
**6**	0.042	0.96	9.58 × 10^−6^	1.47	5.19	12	2.43	0.68
**7**	0.035	0.96	3.23 × 10^−6^	1.18	4.43	10	2.53	0.65
**8**	0.037	0.97	1.83 × 10^−5^	1.73	5.52	16	3.14	0.68
**9**	0.042	0.94	1.89 × 10^−6^	1.21	4.94	19	2.99	0.68
**10**	0.043	0.95	1.32 × 10^−5^	1.53	5.13	12	2.89	0.68
**Ave.**	**0.041**	**0.96**	**9.03 × 10^−6^**	**1.44 ± 0.25**	**5.04 ± 0.49**	**13.5 ± 3.47**	**2.60 ± 0.32**	**0.67 ± 0.02**

**Table 2:** Summary of 10-subject individual average values of RMSE, R2, C0, estimated CRT, the time to reach the stable blood volume, the ratio between the amplitudes A2 and A1; all these features were calculated from the fitting curves (in blue). Additionally shown are the PI and (P–P)_ave_ interval values, which were calculated from the refilling signals (columns 8 and 9). Underfoot temperature and applied and released pressure were obtained from the data logger and the FBG sensor, respectively.

No.	Average Values										
	RMSE	R^2^	C_0_	CRT (s)	Stable Blood Volume (s)	Ratio A2/A1	PI (%)	(P-P)_ave_ Interval (s)	Temp. (°C)	Pressure (kPa)	
										ON	OFF
**P1**	**0.028**	**0.97**	**−1.66 × 10^−5^**	**0.91**	**3.91 ± 1.19**	**9.70 ± 4.24**	**1.21 ± 0.29**	**0.66 ± 0.01**	33.0	105.22	9.71
**P2**	0.035	0.94	−2.42 × 10^−5^	0.82	3.09 ± 0.36	7.90 ± 3.45	1.00 ± 0.33	0.66 ± 0.02	32.8	104.94	12.50
**P3**	0.033	0.96	−2.11 × 10^−5^	0.84	3.33 ± 0.32	9.40 ± 3.57	1.47 ± 0.52	0.70 ± 0.03	31.7	97.34	10.32
**P4**	0.043	0.91	−2.08 × 10^−5^	0.93	3.57 ± 1.14	20.11 ± 5.06	2.45 ± 0.21	0.69 ± 0.04	30.0	94.56	10.33
**P5**	0.021	0.99	2.38 × 10^−6^	1.41	8.35 ± 0.49	38.50 ± 9.63	0.61 ± 0.11	0.76 ± 0.04	28.7	97.03	7.44
**P6**	0.035	0.98	1.22 × 10^−5^	1.82	5.58 ± 0.94	17.63 ± 7.73	2.02 ± 0.16	0.77 ± 0.04	29.0	101.25	9.51
**P7**	0.028	0.97	1.25 × 10^−5^	1.65	6.27 ± 2.45	24.00 ± 5.34	1.21 ± 0.12	0.74 ± 0.04	28.8	102.67	8.56
**P8**	0.041	0.96	9.03 × 10^−6^	1.44	5.04 ± 0.49	13.50 ± 3.47	2.60 ± 0.32	0.67 ± 0.02	32.7	109.62	7.93
**P9**	0.034	0.98	1.58 × 10^−5^	1.89	5.95 ± 0.49	12.70 ± 1.77	1.59 ± 0.20	0.70 ± 0.04	34.1	96.94	8.23
**P10**	0.039	0.97	1.59 × 10^−5^	1.94	6.17 ± 0.88	14.20 ± 4.80	1.89 ± 0.17	0.69 ± 0.03	34.5	102.21	7.32

## Data Availability

The data presented in this study are available on request from the corresponding author.
